# Elimination of the geomagnetic field stimulates the proliferation of mouse neural progenitor and stem cells

**DOI:** 10.1007/s13238-016-0300-7

**Published:** 2016-08-03

**Authors:** Jing-Peng Fu, Wei-Chuan Mo, Ying Liu, Perry F. Bartlett, Rong-Qiao He

**Affiliations:** 1 State Key Laboratory of Brain and Cognitive Science, Institute of Biophysics, Chinese Academy of Sciences, Beijing, 100101 China; 2 Queensland Brain Institute, University of Queensland, Brisbane, QLD 4072 Australia; 3 University of the Chinese Academy of Sciences, Beijing, 100049 China; 4 Alzheimer’s Disease Center, Beijing Institute for Brain Disorders, Capital Medical University, Beijing, 100069 China

**Keywords:** hypomagnetic field, neural progenitor/stem cells, neurosphere, proliferation, stemness, multipotency

## Abstract

Living organisms are exposed to the geomagnetic field (GMF) throughout their lifespan. Elimination of the GMF, resulting in a hypogeomagnetic field (HMF), leads to central nervous system dysfunction and abnormal development in animals. However, the cellular mechanisms underlying these effects have not been identified so far. Here, we show that exposure to an HMF (<200 nT), produced by a magnetic field shielding chamber, promotes the proliferation of neural progenitor/stem cells (NPCs/NSCs) from C57BL/6 mice. Following seven-day HMF-exposure, the primary neurospheres (NSs) were significantly larger in size, and twice more NPCs/NSCs were harvested from neonatal NSs, when compared to the GMF controls. The self-renewal capacity and multipotency of the NSs were maintained, as HMF-exposed NSs were positive for NSC markers (Nestin and Sox2), and could differentiate into neurons and astrocyte/glial cells and be passaged continuously. In addition, adult mice exposed to the HMF for one month were observed to have a greater number of proliferative cells in the subventricular zone. These findings indicate that continuous HMF-exposure increases the proliferation of NPCs/NSCs, *in vitro* and* in vivo*. HMF-disturbed NPCs/NSCs production probably affects brain development and function, which provides a novel clue for elucidating the cellular mechanisms of the bio-HMF response.

**Electronic supplementary material:**

The online version of this article (doi:10.1007/s13238-016-0300-7) contains supplementary material, which is available to authorized users.

## Introduction

Living organisms are exposed to the geomagnetic field (GMF, 35–70 µT) throughout their lifespan. The GMF is well-known for providing navigation information for migrating animals (Gould and Gould 2012; Nathan et al. 2014) or locomotion direction for magnetotactic bacteria (Jogler and Schuler 2009). The effects of disturbed environmental magnetic field have been concerned for long, and that of the hypomagnetic field (HMF, <5 µT), one of the key environmental risk factors for astronauts travelling in outer space, have been considered seriously with the need of manned mission to explore the deep space (Mo et al. 2012a). Recently, wide attention has been drawn on biological roles of the GMF when some animals, even human beings, who were thought to have no magnetic sense, were found to have a potential response to environmental magnetic fields (Lohmann 2010; Gegear et al. 2012). It has been established that the elimination of the GMF has adverse effects on living systems (Mo et al. 2012a). Animals continuously exposed to the HMF condition by shielding or compensatively eliminating the GMF exhibited dysfunction of central nervous system (CNS), with symptoms such as disturbed vocal behavior and circadian activity rhythm in birds (Bliss and Heppner 1976; Jiang et al. 1998); amnesia in chicken (Wang et al. 2002) and *Drosophila* (Zhang et al. 2004); and decreased general activity, altered circadian drinking rhythm and analgesia in mice (Prato et al. 2005; Mo et al. 2015). The HMF also markedly disturbs development processes, evidenced by delayed embryonic and nymphal development in planthoppers (Wan et al. 2014), increased embryo malformation in newt (Asashima and Shimada 1991) and *Xenopus* (Mo et al. 2012b), as well as inhibited early embryogenesis in mice (Fesenko et al. 2010). In particular, abnormalities of the head and spine were marked observed under the HMF condition (Mo et al. 2012b). These data reveal that the GMF is involved in the regulation of the brain functions and development. However, the roles of the GMF on animals are still far from clear, and the cellular mechanisms underlying these effects have not been clearly identified so far .

Neural progenitor/stem cells (NPCs/NSCs) play critical roles in CNS development and maintainence of brain function (Lui et al. 2011; Gage and Temple 2013). Inhibited self-renewal or differentiation of NPCs/NSCs cause either behavioral disorders (Yau et al. 2014; Cameron and Glover 2015) or abnormal development (Merkle and Alvarez-Buylla 2006). It has been reported that exposure to applied magnetic fields affect the growth and fate of NPCs/NSCs (Di Lazzaro et al. 2013). Applied electromagnetic fields (EMFs) inhibit proliferation and promote differentiation of mouse bone marrow mesenchymal stem cells (Wu et al. 2005) and embryonic stem cells (Ventura et al. 2005), and stimulate the maturation and differentiation of cerebellar granule neurons in newborn rat (Lisi et al. 2005). Nakamichi and colleagues showed that exposure to a 100 mT static magnetic field (SMF) reduced proliferation of NPCs in the fetal rat brain (Nakamichi et al. 2009). Thus, disturbance of the GMF could modulate proliferation and differentiation of NPCs/NSCs. Recently, we found that proliferation of human neuroblastoma cells (SH-SY5Y) was accelerated in an HMF (<200 nT) by deep-shielding the GMF (Mo et al. 2013). Therefore, the GMF condition might also be necessary to maintain the homeostasis of NPCs/NSCs, and the HMF may serve as a physical stimulator to the proliferation of NPCs/NSCs. Investigating the HMF effect on the NSC/NPCs would provide useful clues for the cellular mechanism of biomagnetic interactions.

In the present study, we evaluated the growth and differentiation of NPCs/NSCs under the HMF condition (<200 nT). Primary neurospheres (NSs) from the brains of neonatal, young, and adult mice were exposed to either the GMF or the HMF. Our results showed that the growth of the NSs was greatly accelerated in the HMF, and their capacity for self-renewal and multipotency was maintained. Moreover, the number of proliferative cells in the subventricular zone (SVZ) increased in the HMF-exposed adult mouse. Our findings suggest that the NPCs/NSCs can respond to the HMF, and that these bio-magnetic responses could contribute at a cellular level to the GMF’s necessary role in maintaining homeostasis of the CNS.

## Results

### Exposure to the HMF accelerates the growth of primary NSs from the neonatal mouse

**Figure 1:**
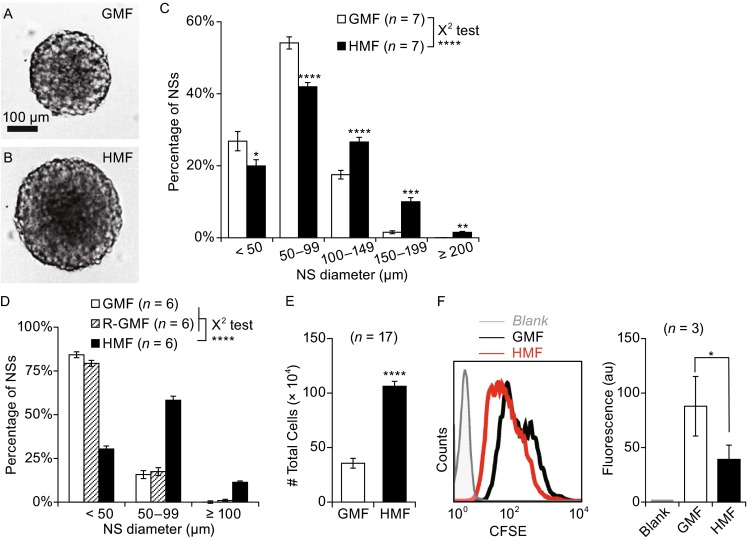
**Exposure to the HMF accelerates the growth of primary NSs from neonatal mouse**. Primary cells from whole brains of P2 mice were seeded in either 60 mm dishes (8.0 × 10^5^ cells/dish for cell counting) or 96-well plates (1000 cells/well for NS counting and size analysis) and exposed to either the GMF or HMF. (A and B) Representative pictures of NSs at day 7. Those grown in the HMF appeared significantly larger. (C) Size distribution of day 7 NSs. A greater number of large NSs were counted in cultures exposed to the HMF. (D) Size distribution of day 6 NSs cultured in the GMF, R-GMF, and HMF conditions. Sizes of NSs in the GMF and R-GMF groups were similar, but smaller than those in the HMF group. (E) Total cell numbers of the day 7 NS cultures were significantly greater in the HMF group, compared to the control GMF group. (F) Cells exposed to the HMF underwent more divisions, as shown by the significantly decreased CFSE fluorescence and lower mean fluorescence. Data are shown as mean ± SEM (C and E) or SD (F). *n* is the number of animals (C–E) and the trials (F) used in the experiments. The *P*-value was calculated using a χ^2^ test for NS size distributions in (C) and (D), a one-way ANOVA for mean comparisons in (E). The two-tailed paired student’s *t*-test in (F) *P < 0.05; **P < 0.01; ***P < 0.001; ****P < 0.0001; n.s. *P* ≥ 0.05

Primary cell suspensions of postnatal day 2 (P2) mouse brains were incubated in either the HMF or GMF environment for 7 days. The morphologies of the NSs exposed to the HMF were similar to those exposed to the GMF; however, the HMF-exposed NSs grew faster and larger (Figs. 1A,  1B and S1). After the final day of exposure to the magnetic fields (day 7), the diameter of each NS was measured, and those incubated in the HMF were found to be significantly larger than those incubated in the GMF (*P* < 0.0001, χ^2^ test; Fig. 1C). Significantly fewer NSs with diameters <100 μm were observed in the HMF (*P* = 0.014, 19.9% ± 1.7%, <50 μm; *P* = 0.0016, 41.9% ± 1.2%, 50–99 μm), when compared to the GMF controls (26.8% ± 2.7%, <50 μm; 54.1% ± 1.7%, 50–99 μm). A greater number of NSs exposed to the HMF had diameters between 100 and 200 μm (*P* < 0.0001, 26.6% ± 1.3%, 100–149 μm; *P* < 0.0001, 10.0% ± 1.1%, 150–199 μm) and also ≥200 μm (*P* = 0.0009, 1.51% ± 0.25%), when compared to the GMF controls (17.5% ± 1.2%, 100–149 μm; 15.2% ± 0.4%, 150–199 μm; 0.025% ± 0.025%, ≥200 μm). As magnetic intensity of the control GMF condition (~15 μT) was lower than the local GMF (~50 μT), we used a reference GMF (R-GMF: 56.6 ± 4.4 μT) in another incubator, as described previously (Mo et al. 2013). The NSs showed no difference in size between the GMF and R-GMF groups at day 6 (*P* = 0.566), while the HMF-exposed NSs were significantly larger than both the GMF and R-GMF groups (*P* < 0.0001, Fig. 1D). These results indicate that the HMF exposure accelerates growth of primary NSs, and the effect is attributed to elimination instead of partial shielding of the GMF.

The NSs formation assay was used to quantify NPCs/NSCs in suspension culture (Louis et al. 2008; Ahmed 2009). At day 7 of primary culture, the number of NSs produced in the HMF (44.0 ± 4.0 NSs per 1000 cells) were very similar with the GMF controls (43.0 ± 4.0 NSs per 1000 cells, *P* = 0.82), which suggests that the HMF-exposure did not activate latent cells or repress proliferation of active cells.

The total number of cells after 7 days of culture in the HMF (10.62 ± 0.45 × 10^5^ cells) was about thrice the number in the GMF control cultures (3.56 ± 0.45 × 10^5^ cells; *P* < 0.0001; Fig. 1E), which indicates promoted proliferation of NPCs/NSCs in the HMF condition and is supported by distributions of NSs sizes above. For further evidences, we used CFSE staining assay to measure cell divisions. When cells undertook divisions, the CFSE was distributed to daughter cells equally and CFSE fluorescence decreased in daughter cells. Results showed that the fluorescence values of the NSs exposed to the HMF were significantly lower than the GMF control cells (*P* = 0.039; Fig. 1F), confirming that a higher amount of cell division occurred when cells were exposed to the HMF. These two assays confirm that the HMF-exposure had accelerated the proliferations of NPCs/NSCs.

### Continued exposure is required for HMF-promoted growth of NSs

**Figure 2:**
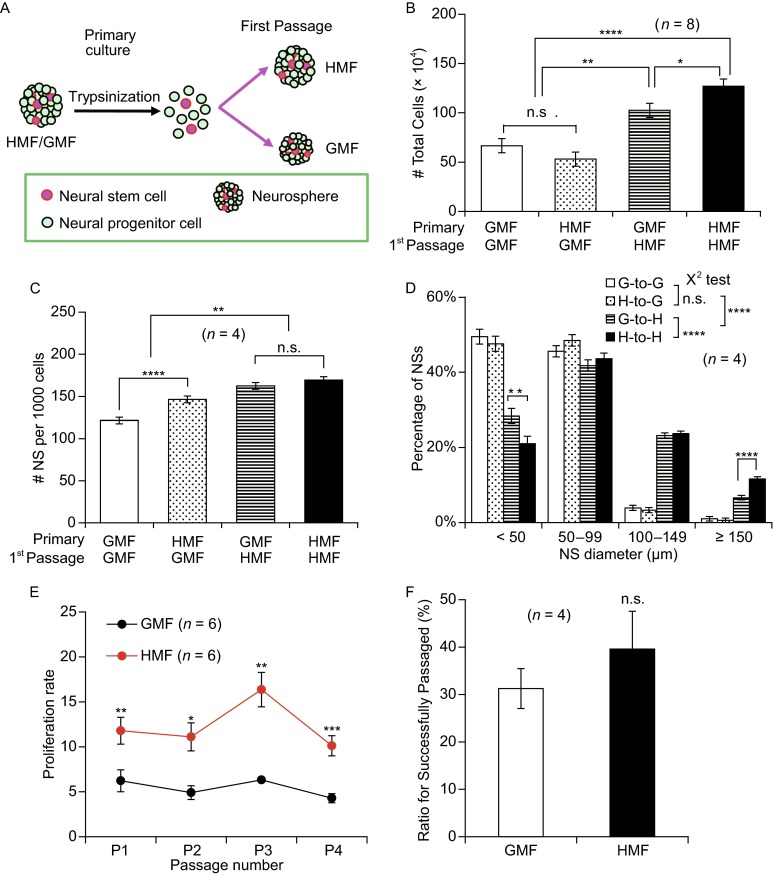
**Continued exposure is required for HMF-promoted growth of NSs**. (A) A schematic of the GMF recovery experiment. Primary cultures from the HMF/GMF condition were trypsinized and exposed to either the HMF or GMF for the first-passage culture in either 60 mm dishes (1.0 × 10^5^ cells/dish for cell counting) or 96-well plates (1000 cells/well for NS counting and size analysis). (B) Numbers of NSs per 1000 seeded cells at day 6. 1000 cells from the HMF-exposed NSs (H-to-G) formed more NSs than the control cells (G-to-G), but the cells growing in HMF condition (G-to-H, H-to-H) formed more cells than these in the GMF condition (G-to-G, H-to-G). (C) Total cell numbers of first passage in the 6th day. More cells were yielded from the NSs growing under the HMF condition than these under the GMF condition, while the cell numbers were similar in the same condition. (D) NS size distributions at day 6. Compared to the NSs in the GMF, the growth of these NSs in the HMF was enhanced. The distributions of NSs size were similar between two GMF groups (G-to-G, H-to-G), while more large NSs and less small NSs were observed in the H-to-H than these in the G-to-H. (E) When HMF-exposed cells from the primary cultures were continuously passaged under the HMF condition (seeded at 1.0 × 10^5^ cells/60 mm dish), they were also seeded in the GMF as controls. NSs maintained a significantly higher proliferation rates in the HMF condition in four passages. (F) The ratio of successfully passaged large NSs (diameter ≥ 150 μm) in single clone culture assays showed no significant difference between the two conditions. *n* is the numbers of experimental trials. Data are shown as mean ± SEM in (B–E), or mean ± SD in (F). *P* values were calculated using a one-way ANOVA in (B–E), and using a χ^2^ test for NS size distribution in (D), and two-tailed paired student’s *t*-text in (F). *P < 0.05; **P < 0.01; *** *P* < 0.001; ****P < 0.0001; n.s. *P* ≥ 0.05

To test whether the accelerated growth of NSs requires continued exposure to the HMF, primary NSs exposed to the GMF or HMF for 7 days were passaged in either the GMF or the HMF, giving four experimental groups: GMF to GMF (G-to-G), GMF to HMF (G-to-H), HMF to GMF (H-to-G), and HMF to HMF (H-to-H) (Fig. 2A). To avoid overgrowth of the NSs that could be stimulated by HMF-exposure in the H-to-H group, the NS assay of the four first-passage cultures were conducted at day 6. The results revealed that the HMF cultures (G-to-H, H-to-H) produced a significantly greater total cell number (*P* < 0.0001), and a greater number of NSs (*P* < 0.01, NSs per 1000 cells) which were also larger (*P* < 0.0001) compared to the cultures exposed to the GMF (G-to-G; H-to-G) (Fig. 2B–D). A greater number of NSs per 1000 cells (*P* < 0.0001) was observed in the H-to-G group (146.40 ± 3.97 NSs per 1000 cells) than the G-to-G group (121.46 ± 3.97 NSs per 1000 cells) (Fig. 2C), confirming that more NSCs/NPCs are produced in the primary culture from the HMF group, which maintain their capacity for self-renewal. The total numbers of cells in the two groups were similar (G-to-G, 6.69 ± 0.71 × 10^5^ cells; H-to-G, 5.32 ± 0.71 × 10^5^ cells; *P* = 0.185) (Fig. 2B), as was the NS size distribution (*P* = 0.936) (Fig. 2D). These results confirm that the growth of the primary NSs in the HMF require continued exposure for accelerated growth of NSs.

### The HMF-exposed NSs maintain their capacity for self-renewal

To evaluate the proliferative capacity of the NPCs/NSCs exposed to the HMF, NSs from primary cultures were continuously passaged in the HMF; a subset of cells was transferred to the GMF condition at each passage to act as controls. The proliferation rate of each passage was recorded on day 6. NSs exposed to the HMF maintained significantly higher proliferation rates (>10 times the seeding cell number) than those returned to the GMF condition (~5 times the seeding cell number) (P1, *P* = 0.0026; P2, *P* = 0.0457; P3, *P* = 0.0024; P4, *P* = 0.0007) (Fig. 2E). The single clone culture assay during the passaging showed that the percentage of large NSs (≥150 μm in diameter) able to be passaged in the HMF (39.6% ± 8.0%) was similar to that in the GMF (31.3% ± 4.2%, *P* = 0.182) (Fig. 2F). The continuous passaging experiment showed that the large NSs from HMF group could be successfully passaged for nine rounds under the HMF or the GMF condition (Fig. S2). These results indicate that NPCs/NSCs can maintain their capacity for self-renewal during exposure to the HMF.

**Figure 3:**
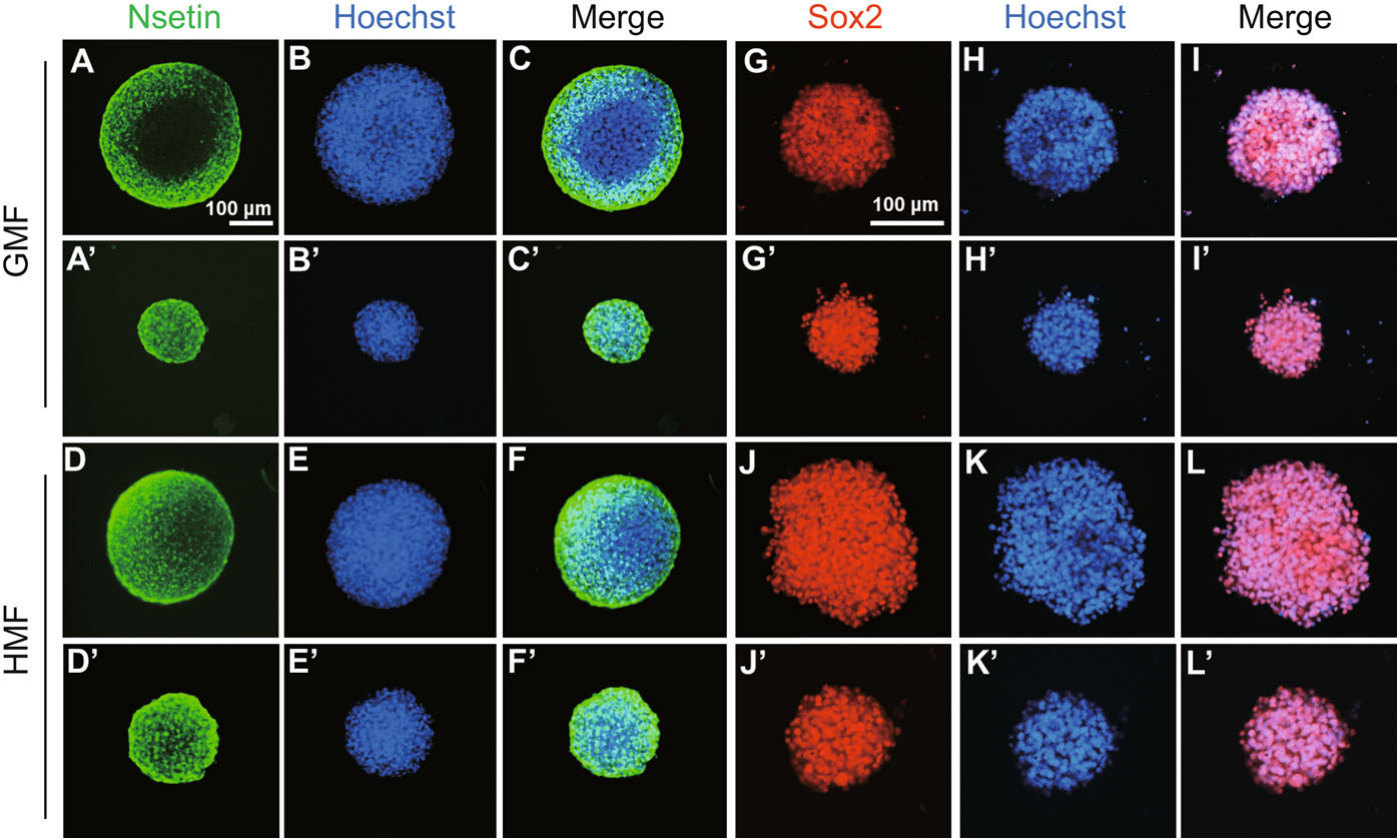
**The HMF-exposed NSs were positive of nestin and Sox2**. Primary cultures of day 7 NSs from P2 mice were collected and immunostained with the neural stem cell markers, nestin (green) and Sox2 (red). Nuclei are stained with Hoechst (blue). Panels show representative large (A–L) and medium-sized (A’ –L’) NSs from the GMF (A–C/A’–C’; G–I/G’–I’) and HMF (D–F/D’–F’; J–L/J’–L’). The NSs of different sizes from both GMF and HMF condition were positive for nestin and Sox2

**Figure 4:**
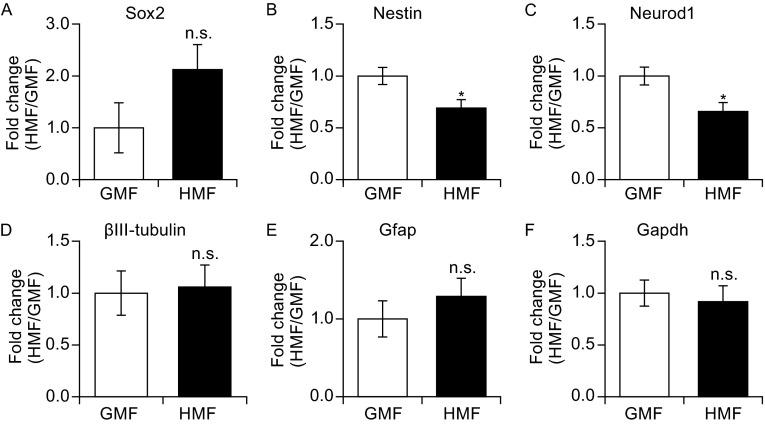
**The HMF-exposed NSs showed altered genes expressions**. When checked expressions of the NSCs markers (*Nestin*; *Sox2*), differentiated neuronal markers (*Neurod1*; *ßIII-tubulin*) and glial marker (*Gfap*), the expression of both *Nestin* (B) and *Neurod1* (C) were significantly down-regulated following exposure to the HMF. Compared to the GMF groups, the *Sox2* had a trend of increase in the HMF group (A).The *ßIII-tubulin* and *Gfap* were found no significant changes (D and E)*. Tubulin 5α* was used as the internal reference gene, and *Gapdh* was used as another internal reference gene (F). Data are shown as mean ± SEM from three independent experiments, six animals per expeiment. *P* values were calculated by one-way ANOVA for mean comparisons. *P < 0.05; n.s. *P* ≥ 0.05

Nestin and Sox2 were used as markers of NPCs/NSCs (Park et al. 2010; Graham et al. 2003). Using an immunofluorescence assay similar to the GMF group, NSs grown in the HMF were found to be both Nestin- and Sox2-positive, confirming that the NSs had kept their ‘stem cell’ identity (Fig. 3A–C/A’–C’; 3G–I/G’–I’ for the GMF; Fig. 3D–F/D’–F’; 3J–L/J’–L’ for the HMF). Interestingly, using a qPCR assay, *Sox2* expression was observed to increase, albeit in a non-significant fashion, in the HMF group to the GMF group (*P* = 0.065; Fig. 4A). *Nestin* expression, on the other hand, had decreased significantly (*P* = 0.029; Fig. 4B). Expression of *Neurod1* was significantly decreased in the NSs exposed to the HMF (*P* = 0.022; Fig. 4C), and no change between these two groups was detected in the expression of the neuronal marker *ßIII-tubulin* (*P* = 0.76; Fig. 4D) nor the glial cell marker *Gfap* (*P* = 0.85; Fig. 4E). No significant change was shown in the expression of *Gapdh*, which was used as an internal reference gene (in addition to *Tubulin 5α*) (*P* = 0.84; Fig. 4F). After 7 days of exposure to the HMF, the expression of both *Cry1* and *Cry2*, putative magneto-sensing genes (Gegear et al. 2012; Xu et al. 2014), was significantly down-regulated (*P* = 0.044 for *Cry1* gene; *P* = 0.048 for *Cry2* gene; Fig. S3), suggesting that the magneto-sensing molecules in the NPCs/NSCs had responded to the HMF. For all the detected genes, the fold-changes in expression between the HMF and the GMF groups were no greater than two. These results indicate that following exposure to the HMF, the culture products maintained the properties of a true stem cell. In the HMF, reduced differentiation was observed, which is consistent with the observed increase in the production of NPCs/NSCs.

### The HMF-exposed NSs maintain their multipotency

**Figure 5:**
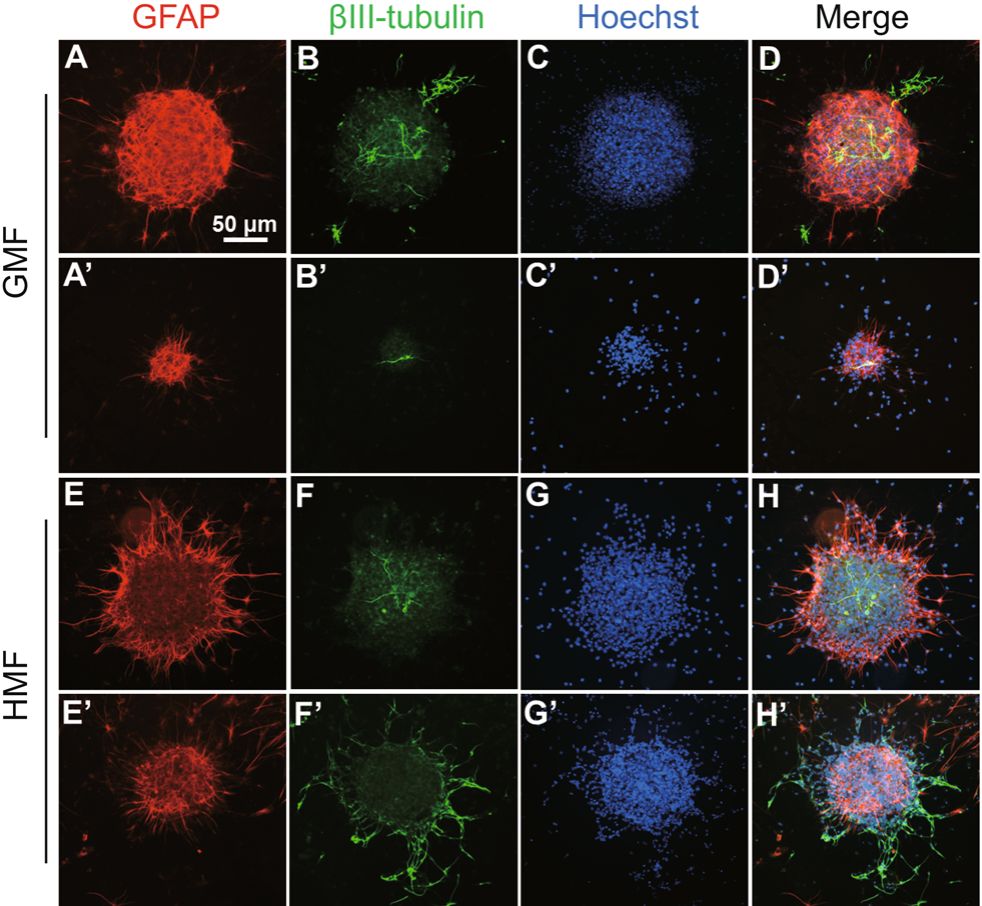
**The HMF-exposed primary NSs can differentiate into astrocytes/glia cells and neurons**. HMF-exposed and GMF primary NSs from P2 mice (day 7) were collected and induced to differentiate under the GMF condition for 5 days. Representative differentiated large (A–H) and middle (A’–H’) size spheres from the GMF (A–D/A’–D’) and HMF (E–H/E’–H’) group show NS cells from HMF condition could differentiate into both neuron (ßIII-tubulin, green) and glial cells (GFAP, red), compared to those from the GMF group. Nuclei were stained with Hoechst (blue)

To test whether NSs exposed to the HMF retained their multipotency, these NSs of different sizes from primary cultures of either the GMF or HMF groups were collected and made a differentiation assay in the GMF condition, and an immunofluorescence assay was subsequently used to detect their differentiation states. Both GFAP-positive and ßIII-tubulin-positive cells were observed in the HMF-exposed NSs as well as in those exposed to the GMF (Fig. 5A–D/A’ –D’ for the GMF; Fig. 4E–H/E’–H’ for the HMF), indicating that the NSs retained their ability to differentiate into both neurons and glial cells despite exposure to the HMF.

### The HMF counteracts the negative effects of the removal of growth factors on NS growth

**Figure 6:**
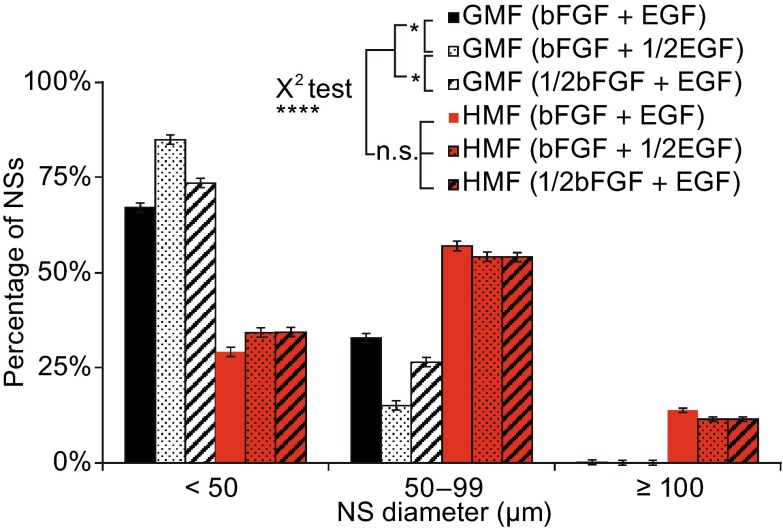
**Exposure to the HMF counteracts the negative effects of the removal of growth factors on NS growth**. Primary cells were seeded in complete medium, EGF-halved (1/2EGF) or bFGF-halved (1/2bFGF) medium, and cultured in the GMF/HMF condition for 6 days. Compared to cells in the complete medium, NSs significantly decreased in size when the concentration of EGF was halved in the GMF but not in the HMF, and the NS size of the three groups in HMF were significantly larger than those in the GMF. Three trials were completed with 18 repeated wells in each trial. Data are shown as mean ± SEM. *P* values were calculated using a χ^2^ test for NS size distribution comparison. *P < 0.05; ****P < 0.0001; n.s. *P* ≥ 0.05

The growth factors bFGF and EGF are critical in the maintenance of NSs growth as well as the production of NSCs (Ciccolini and Svendsen 1998; Azari et al. 2010). To assess the effect of the HMF on the growth factor sensitivity of NS cells, the concentration of either EGF or bFGF in the culture medium was halved (10 ng/mL EGF or 5 ng/mL bFGF), and an NS assay conducted at day 6. Very few NSs with a diameter of >50 μm were observed in both the HMF and GMF primary cultures when both EGF and bFGF were reduced (Data not shown). As shown in Figure 6, when compared to those cultured in normal media (20 ng/mL EGF and 10 ng/mL bFGF), in the reduced EGF medium, the percentage of NSs with a diameter between 50–100 μm was significantly smaller in the GMF-exposed groups (*P* = 0.012), and a trend toward a decrease was observed in bFGF-halved culture media (*P* = 0.624). NSs with diameters ≥100 μm were observed almost exclusively in the HMF; very few were observed in the GMF cultures. However, no significant differences were observed in the HMF-exposed groups in the reduced growth factor medium and in the normal medium, which indicates that the HMF still stimulates NSs growth under shortage of growth factor condition. These results indicate that exposure to the HMF can counteract the negative effects on NS growth induced by shortage of exogenous growth factors.

### The HMF promotes the proliferation of NPCs/NSCs from the adult mouse brain

**Figure 7:**
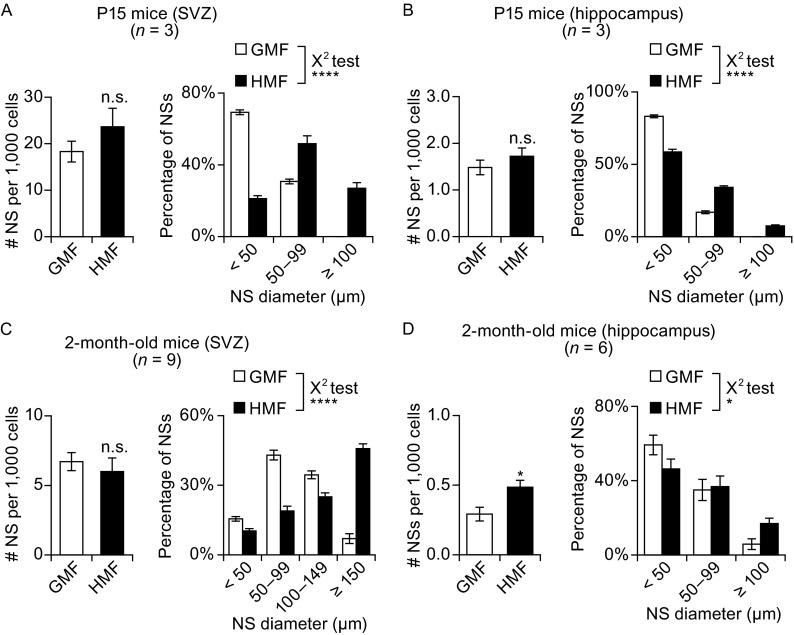
**The HMF promotes the proliferation of NPCs/NSCs from the adult mouse brain**. SVZ and hippocampal tissues from young (P15) (A and B) and adult (2-month-old) mice (C and D) were collected and cultured in either the HMF or GMF. The number of NSs per 1000 primary cells (left panels) and the NSs size distributions (right panels) were determined. A greater number of large NSs were observed in young and adult SVZ cultures, as well as young and adult hippocampal cultures. The number of NSs was significantly greater than the HMF only in the adult hippocampal cultures, but was found with no changes in other groups. *n* is the number of animals. Data are shown as mean ± SEM. *P* values were calculated using a one-way ANOVA for mean comparisons and using a χ^2^ test for NS size distribution comparison. *P < 0.05; ****P < 0.0001; n.s. *P* ≥ 0.05

In mature mammalian brains, NPCs/NSCs are mainly restricted to the SVZ (Golmohammadi et al. 2008) and the hippocampus (Bull and Bartlett 2005). The growth of primary NSs from the SVZ and hippocampus in young (P15) and adult (2-month-old) mice was compared following exposure to either the HMF or GMF. Primary SVZ NS cultures from both young and adult mice were found to have similar numbers of NSs irrespective of the magnetic field to which they were exposed (Fig. 7A and 7C, left panels). A greater number of large NSs (diameter ≥ 100 μm) was observed following exposure to the HMF in both groups (P15, *P* < 0.0001; 2-month-old, *P* < 0.0001) (Fig. 7A and 7C, right panels).

Compared to the SVZ groups, the overall number of NSs formed from the primary hippocampus was very low in both the GMF and HMF conditions (<1.5 NSs per 1000 cells) (Fig. 7B and 7D, left panels). No significant difference was observed between the HMF and GMF groups in NSs numbers per 1000 cells from the P15 mice (Fig. 7B, left panel), but the 2-month-old mice showed a significantly greater number of NSs per 1000 cells following exposure to the HMF (0.29 ± 0.05 for the GMF group, 0.48 ± 0.05 for the HMF group; *P* = 0.023; Fig. 7D, left panel). As observed in the SVZ NSs, a greater number of large hippocampal-derived NSs (P15, *P* < 0.0001; 2-month-old, *P* = 0.046) were present in the NS cultures exposed to the HMF for both age groups (Fig. 7B and 7D, right panels). These results indicate that HMF exposure triggers an acceleration of NS growth in primary cultures from both young and adult mice brains, and promotes formation of NSs from the adult hippocampus.

### Exposure to the HMF increases cell proliferation in the adult mouse brain

**Figure 8:**
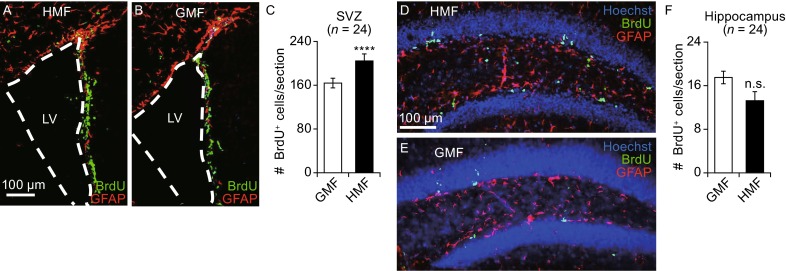
**HMF exposure promotes cell proliferation in the adult mouse brain**. Adult male mice (4 to 6-week-old) were reared in either the HMF or GMF conditions for 30 days. Representative images of the SVZ are shown in panels (A) and (B), and the hippocampus in (D) and (E). Proliferative cells were immunostained with anti-BrdU antibody (green) and glia cells with anti-GFAP antibody (red). Nuclei were counterstained with Hoechst (blue). White dash lines outline the edge of the lateral ventricles (LV). (C and F) show number of BrdU-positive cells per section from the SVZ (C) and hippocampus (F). Exposure to the HMF increased the number of proliferative cells in the SVZ but not the hippocampus. *n* indicates the number of animals from at least three independent experiments. Data are shown as mean ± SEM. *P* values were calculated using a one-way ANOVA for mean comparisons. ****P < 0.0001; n.s. *P* ≥ 0.05

To investigate whether exposure to the HMF has a positive effect on proliferation of NPCs/NSCs *in vivo*, adult mice were reared in an environment in which they were exposed to the HMF for 30 days. As shown in Fig. 8, BrdU-positive cells were observed in sections of the SVZ after mice had been exposed to both the HMF and GMF conditions (Fig. 8A and 8B). Exposure to the HMF resulted in a significant increase in the total numbers of BrdU-positive cells (*P* < 0.0001; Fig. 8C), which suggests a greater degree of proliferation in the SVZ. BrdU-positive cells were also detected in the hippocampus (Fig. 8D and 8E), but no significant differences between the two groups were observed (Fig. 8F; *P* = 0.059). These data demonstrate that exposure to the HMF promotes the proliferation of adult NPCs/NSCs in the SVZ, but not the hippocampus, *in vivo.*

## Discussion

Our results provide the first evidence that elimination of the GMF affects the growth of NPCs/NSCs, and that the GMF is required for maintaining the homeostasis of stem cells in the CNS. Following exposure to the HMF condition: (1) The proliferation of NPCs/NSCs from newborn (P2), young (P15), and adult mice (2-month) is accelerated *in vitro*; (2) these NPCs/NSCs were positive for NSC markers (Nestin and Sox2), and could be continuously passaged and differentiated into neurons and astrocyte/glial cells; (3) the HMF-enhanced NS growth could be maintained during continuous passages and restored by the GMF recovery; (4) the number of proliferative cells in adult SVZ were increased *in vivo*. These results indicate that NPCs/NSCs in the CNS can respond to the HMF.

Previously, the HMF exposure has been found to cause abnormal cognitive behaviors and disrupt embryonic development (Wang et al. 2002; Zhang et al. 2004; Asashima and Shimada 1991; Mo et al. 2012b; Fesenko et al. 2010), but the potential biological mechanism is rarely been investigated. The proliferation, differentiation, and quiescence of NPCs/NSCs are under strict control and kept in delicate balance in embryonic as well as in adult brains (Simons and Clevers 2011; Stine and Matunis 2013). Excessive proliferation of stem cells would lead to stem cell exhaustion and aging (Orford and Scadden 2008; Oh et al. 2014), probably resulting in abnormal development and dysfunction of the CNS. Hyper-proliferation of NPCs/NSCs was reported to cause brain overgrowth and autism-associated behaviors (Nordahl et al. 2013; Le Belle et al. 2014), and decreased neurogenesis in affected animals led to interrupted learning and memory (Cameron and Glover 2015). According to our results, the HMF accelerates the proliferation of NPCs/NSCs *in vivo* (Fig. 8), which could break the balance of cell activities in CNS. Besides, HMF induced pro-proliferation of NSCs could also result in hyper-proliferation of NPCs/NSCs in brains. Thus, our study suggests that disturbed NPCs/NSCs activities under the HMF condition could be a reason for the bio-HMF effects on animal development and behaviors.

Usually, NSs formed by NPCs are smaller than those formed by NSCs under normal culture condition (Louis et al. 2008; Ahmed 2009). The growth of both small-sized and large-sized NSs from embryonic brains were significantly accelerated by the HMF-exposure (Figs. 1, 2 and 7), indicating a common pro-proliferation effect on both NPCs and NSCs. In addition, the self-renewal capacity is limited in NPCs but not in NSCs so that NSCs can be enriched during continuous passaging. The pro-proliferative effect on NSs in the HMF maintained during the continuous passages (Figs. 2E and S2), proving the growth of NSCs is accelerated in the HMF. On the other hand, both NSCs and NPCs are present in the adult SVZ (Golmohammadi et al. 2008), but only NPCs and very few NSCs are found in the hippocampus (Bull and Bartlett 2005). Our results showed that the growth of NSs from young and adult hippocampus tissues were enhanced in the HMF (Fig. 7), which confirmed that the exposure to HMF has a pro-proliferative effect on NPCs. However, the response of NPCs and NSCs to the HMF might be different. The *in-vitro* NS assay and *in-situ* BrdU assay showed that the proliferation of NSCs/NPCs from the adult SVZ was greatly enhanced after exposure to the HMF condition (Figs. 7C, 8A–8C); however, the effect on adult hippocampal NPCs was rather low *in vitro*, and no significant change was observed *in vivo* (Figs. 7D, 8D–8F). These results suggest that NPCs are less sensitive to the HMF exposure than NSCs, though the proliferation of both NSCs and NPCs can be accelerated in the HMF.

The pro-proliferation effect of the HMF is sustainable and reversible. When NSs were exposed to the HMF for four continuous passages, higher proliferation rate of the HMF group was maintained (Fig. 2E), and cell numbers increased continuously during the nine passage rounds (Fig. S2), which confirmed that the HMF effect sustained during the exposure. The numbers of cells were increased in all four passages and theoretically yielded a total of 1.88 ± 0.45 × 10^10^ cells, 26.3 times that the GMF group (8.79 ± 3.17 × 10^8^). Since huge amount of cells are required for stem cell transplantations (Tsukamoto et al. 2013), our study suggests that HMF exposure could serve as a novel method to produce donor cells for stem cell therapy. Meanwhile, the growth of the HMF-exposed NSs dropped to the same level as the GMF controls when they returned to the GMF condition; the reversible HMF effect on NS growth is consistent with the previously reported HMF effects on animal behavior. HMF-induced (10 generations of continuous HMF exposure) amnesia in *Drosophila* has been shown to be recoverable after six consecutive generations in the GMF (Zhang et al. 2004). Since HMF is one of the environmental factors of outer space, GMF recovery would be a good option to minimize the HMF-related health risks to astronauts participating with long-term and long-distance space missions (Mo et al. 2014).

Interestingly, although the HMF-exposed NSs maintain self-renewal and differentiation capacity, the transcription of *Nestin,* the common NSC marker, and *Neurod1*, the terminal neuronal differentiation marker (Cho and Tsai 2004), were downregulated in the HMF-exposed NSs compared with that in the GMF (Fig. 4B and 4C). The decrease of the transcription of *Neurod1* is consistent with the enrichment of NSCs and an overall decrease of differentiation level in the HMF-exposed cells. The reduced transcription of *Nestin* suggests a contrary direction of cell fate, as *Nestin* is required for proper self-renewal and survival of NSCs (Park et al. 2010), and is down-regulated after differentiation (Namiki et al. 2012). Thus, the molecular property of the HMF-exposed NPCs/NSCs might be changed, although their ‘stemness’ identity is still maintained. However, the expressions of *Gfap* and *ßIII-tubulin* were not changed in the HMF-exposed NSs after the 7 day culture, as compared with the GMF control group (Fig. 4D and 4E), indicating that the pluripotency of the HMF-exposed cells to neuronal cells and astrocyte/glia was maintained. The differentiation assay confirmed that the HMF-exposed NSs could differentiate into neurons and astrocytes/glia (Fig. 5). Therefore, the HMF-exposure maintains ‘stemness’ of NSs, but might reduce their potency of neuronal differentiation. It would be interesting to examine the cell properties after exposure to the HMF to investigate whether this magnetic field could be applied to regulate the fate of NSCs.

Our data showed that the NSs under the HMF exposure could tolerate the shortage of EGF or bFGF (Fig. 6). EGF and bFGF play a pivotal role in maintaining the self-renewal capacity of NPCs/NSCs in primary culture, and their signals were transduced via EGF receptors (EGFR) and bFGF receptors (bFGFR). Recently, it has been established that EGFR could serve as the mediator of the biomagnetic effects. A 0.4 mT power frequency magnetic field could induce clustering of the EGFR (Jia et al. 2007) and act as a stimulation factor, similar as the EGF, to activate the EGFR signaling (Wu et al. 2014). Since identical concentrations of EGF and bFGF were provided in the GMF-control and HMF-exposed groups under the normal and growth factor shortage incubation situation, it is probably that the HMF had elevated the activity or signal transduction efficiency of the growth factor receptors, EGFR and bFGFR in the NSs. Additionally, based on our *in-vivo* results, the levels of EGF and bFGF in the brain of HMF-exposed mice are also worth to be measured in the further study, to extend our understanding on the HMF effects on the growth of NSCs/NPCs. Moreover, ROS is involved both in the regulation of EGFR signaling (Carreira et al. 2010) and in the biological responses to the HMF (Martino and Castello 2011; Portelli et al. 2012; Fu et al. 2016) and weak EMF (Usselman et al. 2014; Castello et al. 2014). Recently, we found that the HMF could increase cellular ROS levels in primary skeletal muscle cells (Fu et al. 2016); however, no significant changes on oxidative stress signaling were detected in the HMF-exposed mice (Ding et al. 2014). Therefore, the role of ROS in the HMF effects is complicated and further investigation is necessary to elucidate the function of ROS in the HMF-enhanced NPCs/NSCs proliferation.

In terms of the molecular mechanism of the HMF effects, a number of recent publications have provided evidence to support the role of *Cry* genes (*Cry1* and *Cry2*) as the key molecules in the bio-magnetic interaction in avian, *Arabidopsis*, *Drosophila* and human cells (Mo et al. 2013; Xu et al. 2014; Gegear et al. 2012). Although Crys are photoreceptors and the radical pair generated by photo-chemical reaction in Crys plays a key role in its magnetosensitive function (Biskup et al. 2009), Crys can also generate radical pairs by the light-independent dark reoxidation of the flavin cofactor (Müller and Ahmad 2011). Recently, it has been shown that Cry could form a magnetic protein complex with MagR and sensing the direction of the applied static magnetic field, *in vitro* (Qin et al. 2016). Therefore, Crys would probably acting as an important molecule in the sensation of the elimination of the GMF under the dark condition such as in a cell incubator. Accumulating evidence has shown that cells and tissues incubated in the HMF exhibited observable cellular responses which were light-independent (Fesenko et al. 2010; Mo et al. 2013; Martino and Castello 2011; Fu et al. 2016; Martino et al. 2010; Mo et al. 2016). Our previous study has screened HMF-responding genes from neuroblastoma cells and verified that Cry2 was significant up-regulated after an 18 h short-term HMF exposure and down-regulated after a 48 h continuous HMF exposure (Mo et al. 2014b). Reduced mRNA levels of both *Cry1* and *Cry2* were detected in the HMF-exposed NSs (Fig. S3). Thus, Crys in non-photo-sensing organs, such as NPCs/NSCs would also play a role in magnetosensation. Down-regulated *Cry1* expression in cells was found to cause accelerated growth rate in primary fibroblasts (Destici et al. 2011), so it is probable that the HMF-induced *Cry* down-regulation has contributed to the accelerated NS growth. Investigating the role of *Cry* genes, would help to explain how stem cells respond to the HMF at the molecular level.

In conclusion, our research proves that the GMF is required for the maintenance of stem cell homeostasis. Exposure to the HMF accelerates the proliferation of NPCs/NSCs *in vitro* and *in vivo,* but the self-renewal and pluripotency capacity of NPCs/NSCs is maintained. Disturbed NPCs/NSCs growth in brains would probably contribute to abnormal development and altered behaviors in HMF-exposed animals. Furthermore, stem cell culturing in the HMF, such a physical approach which stimulates the growth of NPCs/NSCs non-invasively, has great potency for clinical application in stem cell therapy.

## Materials and methods

### The HMF conditions

The HMF condition for cell culture was established as described (Mo et al. 2013). The samples exposed to the HMF were cultured in a magnetic shielding chamber with a residual magnetic field <200 nT. The GMF control samples were cultured on a plastic shelf outside the magnetic shielding box with a local magnetic field of 15.1 ± 2.2 μT. The other conditions inside and outside the chamber were almost identical.

The HMF environment for animal rearing was provided by a 3-axis Helmholtz coils system (HCS) as described (Mo et al. 2015). The HMF-exposed animals were reared in a residual magnetic field of 0.029 ± 0.029 μT (center) and 0.55 ± 0.3 μT (average value); the control animals were reared on a wooden table (49.88 ± 1.82 μT average value), 1.5 m away from the HCS, in the same room (Table S1). The alternating magnetic fields (AMFs) were measured using a CCG-1000 induction alternative magnetometer (National Institute of Metrology, Beijing, China) and the predominant AMF frequencies were checked from the output of signal using a Textronics TDS 2014 digital real-time oscilloscope (Tequipment.NET, Long Branch, NJ, USA). The AMFs of the HCS and the GMF control environments were identical.

### Animals

C57BL/6 mice were provided by the animal experiment center of the Institute of Biophysics (IBP). P2 (male/female), P15 (male), and 2-month (male) littermate naïve mice were used for primary NS cultures. 4–6 week male mice were used for the *in vivo* assay. All animal experiments were approved by the Animal Care and Use Committee at the IBP, Chinese Academy of Sciences (CAS) (authorized No.: SYXK2014-31) and carried out in accordance with the national guidelines for the care and use of laboratory animals.

For the HMF/GMF exposure assay, animals were firstly reared on the GMF for 7 days to adapt to the environment, and then randomly grouped as four mice per standard “shoebox” cage. The cages in the HMF group were aligned as described (Prato et al. 2005). Animals were reared under a 12 h/12 h light/dark cycle. Daily magnetic field fluctuation was recorded, and the room temperature and humidity was maintained at 22 ± 1°C and ~40%–60%, respectively (Fig. S4).

### Primary neurosphere (NS) culture

The whole brain, SVZ and hippocampus of mice were obtained as described previously (Walker et al. 2008). Whole brain P2 mouse or adult SVZ samples were enzymatically digested (0.25% trypsin and 0.025% EDTA in PBS) for 7–10 min at 37°C, and the hippocampus were digested using papain [1 mg/mL papain (Sigma-Aldrich, St. Louis, MO) and 0.5 mg/mL DNase I in L-15 medium (Invitrogen/molecular probe, Grand Island, NY)] for 10–15 min at 37°C. The mixtures were then mechanically triturated and filtered through 40 μm sieves (BD Bioscience, San Jose, CA). Cells were collected by centrifugation and resuspended in NS culture media (NSA) [DMEM/F12 (Invitrogen) supplemented with 10% proliferation supplement (Stem Cell Technologies, Vancouver, British Columbia, Canada), 2% bovine serum albumin (BSA) (Roche, Basel, Switzerland), 2 μg/mL heparin (Sigma-Aldrich), 10 ng/mL fibroblast growth factor 2 (bFGF) (Roche), and 20 ng/mL epidermal growth factor (EGF) (BD Bioscience)]. Then the primary cells were plated in a 96-well plate (1000 cells/well with 200 μL NSA medium) for NS counting and size analysis, or in a 60 mm dish (8.0 × 10^5^ cells/dish with 4 mL NSA medium) for cell counting. The number and size of mature NSs (diameter ≥ 40 μm) were determined with an inverted microscope.

### NS passaging

The cultured NSs were collected and passaged at day 7. The NSs were washed with PBS, trypsinized and then mechanically triturated into dissociated cells in NSA medium. After cell counting (N_total d7_), the cells were seeded at a density of 1000 cells per well with 200 μL NSA medium in a 96-well plate for NS counting and size analysis, or 1.0 × 10^5^ cells (N_seed d0_) per 60 mm dish with 4 mL NSA medium for counting total cell numbers (N_total d7_). Cell proliferation rate (R) was calculated as: R = N_total d7_/N_seed d0_. The theoretical total number of cells obtained at a certain passage (N_Pi_) was calculated as: N_Pi_ = N_seed P1_*R_P1_*R_P2_*…*R_Pi_.

For single clone culture, individual large NS (diameter ≥ 150 μm) was collected and trypsinized, as described above. All cells were seeded in a 6-well plate with 2 mL NSA medium per well. Successful cultures were determined when large NSs (diameter ≥ 150 μm) were observed at day 7. At least six NSs were used in each trial.

### CFSE staining

Cell division in the primary NS was measured with Carboxyfluorescein Diacetate Succinimidyl Ester (CFSE) (Sigma-Aldrich) staining as described (Quah and Parish 2010). Primary cells from P2 mouse brain were stained with 25 μmol/L CFSE (10^7^ cells/mL) for 20 min at 37°C. After two washes with PBS (with 0.1% BSA), CFSE-stained cells were seeded in a 60 mm dish and incubated in the HMF or GMF condition, as described above. NSs were collected and trypsinized at day 7, CFSE fluorescence was measured using a FACS Calibur flowcytometer (BD Bioscience) and analyzed with the Cell Quest Pro software. The cells that were not stained with CFSE were used as blank control (Blank).

### Quantitative real-time PCR (qPCR)

Total RNA of primary NSs was extracted using an RNA extraction kit (QIAGEN, Hilden, Germany). cDNA samples were synthesized using an EasyScript First-Strand cDNA Synthesis SuperMix (Transgen Biotech, Beijing, China). The gene-specific primers (Supplementary Table S2) were designed by PrimerBank (Wang et al. 2012). A TransStart Green qPCR SuperMix UDG kit (TransGen Biotech) was applied to prepare the qPCR samples, which were run in triplicate on a Rotorgen Q real-time PCR cycler (QIAGEN). Thermal cycling was performed at an initial UDG incubation step at 50°C and a UDG inactivation step at 94°C, and then subjected to 45 cycles of 15 s denaturing at 95°C, 30 s at annealing temperature, and a 30 s extension at 72°C. Quantitative gene expressions were referenced to Tubb5 and normalized to the GMF samples.

### NS differentiation assay

NS differentiation was performed as described (Golmohammadi et al. 2008) under the GMF condition. Coverslips were pre-coated with 15% poly-ornithine (Sigma-Aldrich) and 2% laminin (Invitrogen) at 37°C overnight and then washed six times with PBS. At day 7, mature NSs (10–20 NSs per coverslip) were transferred onto the coverslip and incubated in 2 mL differentiation medium (90% DMEM/F12 and 10% proliferation supplement) in each well. After 5 days of incubation, the NSs became adhered to the coverslips.

### Immunofluorescent staining

Immunofluorescent staining of mature or differentiated NSs was performed as described (Golmohammadi et al. 2008). The NSs were fixed with 4% paraformaldehyde (Amresco, Solon, OH) for 30 min at RT. After one PBS wash, they were blocked in blocking solution [PBS with 0.1% triton100 (Amresco), 5% FBS, and 5% goat serum (ZSGB-Bio, Beijing, China)] for 60 min at 37°C. After that, the NSs were firstly stained with primary antibody and then with secondary antibody in blocking solution, the nuclei were stained with 10 μg/mL Hoechst (1:1000; Beyotime, Jiangsu, China). Primary antibodies: mouse-anti-nestin (1:200; 307501, R&D, Minneapolis, MN), rabbit-anti-SOX2 (1:200; L1D6A2, Cell Signal Technology (CST), Boston, MA), mouse-anti-GFAP (1:200; GA5, CST) and rabbit-anti-ßIII-tubulin (1:200; D65A4, CST); secondary antibodies: Alexa Fluor 568 donkey-anti-mouse (1:1000; Invitrogen/molecular probe) and Alexa Fluor 488 donkey-anti-rabbit (1:1000; Invitrogen/molecular probe). The coverslips were then mounted using GVA mounting medium (ZSGB-Bio).

### BrdU assay

Animals were administered with BrdU (10 mg/mL in physiological saline; Sigma-Aldrich) twice per day by intraperitoneal injection at a dose of 350 mg per kilogram body weight, 3 days before sacrifice. After ether anesthesia, animals were transcardially perfused with 50 ml normal saline and 50 mL 4% PFA. The brains were fixed in 4% PFA overnight at 4°C, then cryoprotected in 30% sucrose and embedded in embedding medium (Tissue-Tek; Sakura Finetek, Torrance, CA). Transverse sections were cut using a cryostat (10 µm) (Leica, Wetzlar, Germany). For immunofluorescence staining, sections were re-hydrated (three PBS washes and 2 mol/L HCl for 1 h at RT). After four PBS washes, sections were treated with blocking solution (PBST and 10% goat serum) for 1 h at RT, then incubated in sheep-anti-BrdU antibody (ab1893, Abcam, Cambridge, MA) solution (1:200 in blocking solution) at 4°C overnight. After three PBS washes, sections were incubated in Alexa Fluor 488 donkey-anti-sheep antibody (1:300, Invitrogen/Molecular probe) for 2 h at RT. Following three PBST washes, the nuclei were stained with Hoechst (10 µg/mL) at RT for 20 min. The sections were mounted with GVA mounting medium. The number of BrdU-positive cells was determined from five consecutive sections (Wojtowicz and Kee 2006).

### Statistical methods

Each experiment was repeated at least twice in triplicate each time, if not otherwise specified. A one-way ANOVA was used for mean comparison. A χ^2^ test was used for the comparison of NS size distribution p < 0.05.

### Microscopy

Phase contrast images of alive NSs in the culture medium were taken at RT using an inverted microscope with UPlanFl 10×PH/0.3 objective lens (Olympus IX71, Japan) and a cooled EMCCD (Andor iXon DV897, UK, 512 × 512 pixels).

Fluorescent images of immunostained NSs and sections were taken using a fluorescent microscope with Plan APO 10×/0.45 or 20×/0.75 objective lens (Nikon FXA, Japan), and a cooled CCD (Olympus DP71, Japan, 1360x1024 or 4080× 3072 pixels). G-2A (EX510-560/DM575/BA590), B-2A (EX450-490/DM505/BA520), UV-2A (EX330-308/DM400/BA420) filters (Nikon) were applied for Alexa Fluor 568, Alexa Fluor 488 and Hoechst, respectively. Images were merged with Adobe Photoshop CS4.

## Electronic supplementary material

Below is the link to the electronic supplementary material.

Supplementary material 1 (PDF 642 kb)
