# Supplementary material for: Elimination of the geomagnetic field stimulates the proliferation of mouse neural progenitor and stem cells
Source: Protein Cell. 2016 Aug 3;7(9):624–37. doi: 10.1007/s13238-016-0300-7 (PMC5003790; doi:10.1007/s13238-016-0300-7)
Supplement: Supplementary file 1 — Supplementary material 1 (PDF 642 kb) [file 13238_2016_300_MOESM1_ESM.pdf]

Elimination of the Geomagnetic Field Stimulates the Proliferation of Mouse Neural Progenitor and Stem Cells

Jing-Peng Fu[1,3,†], Wei-Chuan Mo[12,†], Ying Liu[1,3,*], Perry F. Bartlett[2], and Rong-Qiao He[1,3,4,*]

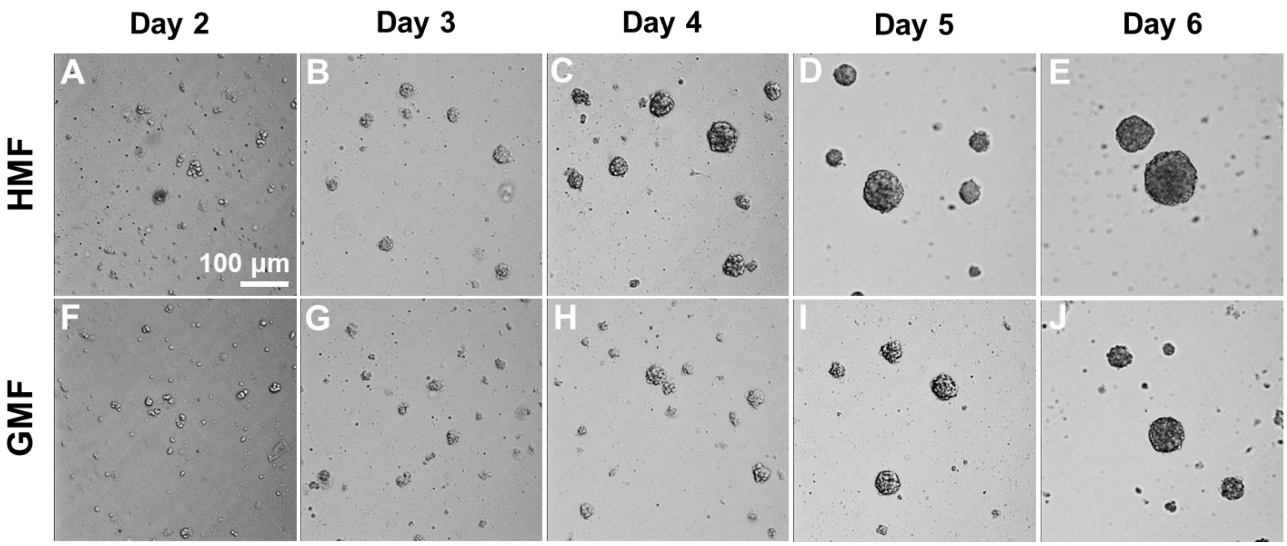

Supplementary Figure S1. Representative images of NSs from day 2 to day 6 cultures. **A–E:** HMF-exposed NSs. **F–J:** GMF-control NSs. The morphologies of the NSs exposed to the HMF were similar to those exposed to the GMF but the size of the HMF-exposed NSs appeared to be larger.

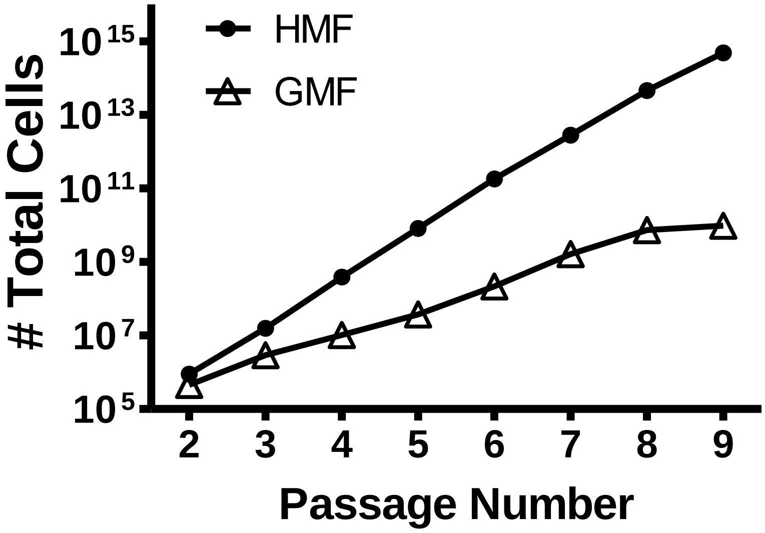

Supplementary Fig. S2. Large NSs (diameter > 150 μm) from the HMF-exposed or GMF control primary cultures could be continuously passaged for 9 passages from an independent experiment.

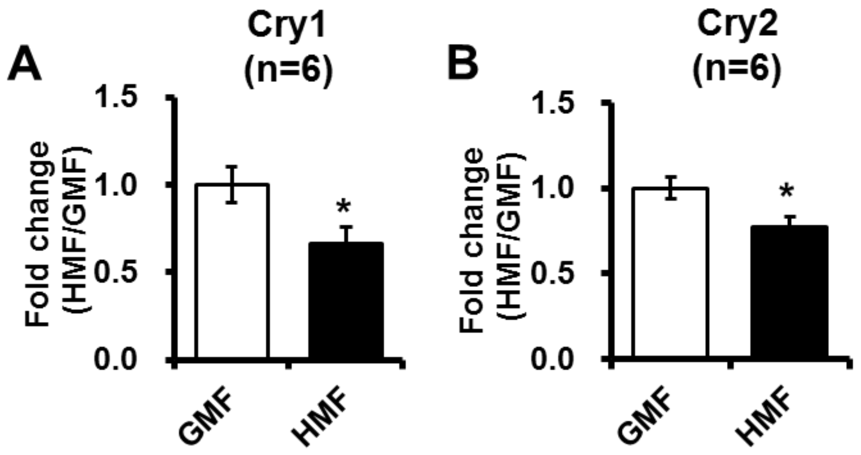

Supplementary Figure S3. Expression of the magneoreceptor genes *Cry1* and *Cry2* at the mRNA level, measured using qPCR. After 7 days exposure in the HMF condition, both *Cry1* and *Cry2* were down-regulated. n is the number of animals from three independent experiments. Data are shown as mean ± SEM. p values were calculated using a one-way ANOVA for mean comparisons. *$p<0.05$.

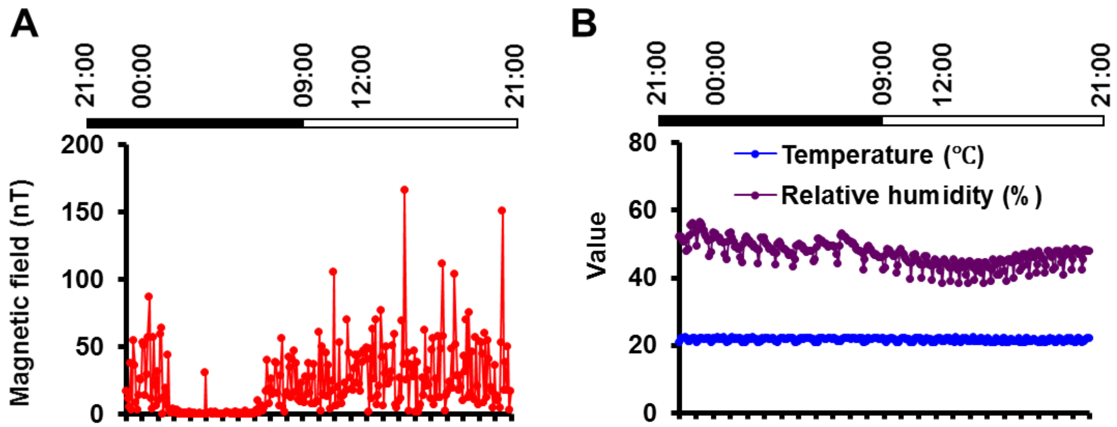

Supplementary Figure S4. A representative daily records of the experimental environment. **A**: The daily fluctuation of the residual magnetic field at the center of the HCS at 5 min intervals. **B**: The daily record of the relative humidity and temperature in the experimental room at 5 min intervals.

Supplementary Table S1. Magnetic field conditions for animal assay (Mo et al. 2016) (Unit: μT).

	**|B|**	**Frequency** (Hz)	**|B_x|**	**|B_y|**	**|B_z|**
**The static magnetic field**					
HMF_{center}	0.029 ± 0.029	/	0.009 ± 0.018	0.007 ± 0.010	0.023 ± 0.025
$HMF_{avreage}$	0.55 ± 0.30	/	0.22 ± 0.16	0.37 ± 0.29	0.22 ± 0.22
GMF_{center}	46.3 ± 1.24	/	25.8 ± 1.29	0.85 ± 0.56	38.5 ± 0.64
$GMF_{average}$	49.88 ± 1.82	/	30.62 ± 3.94	4.51 ± 2.38	38.85 ± 1.75
**The alternating magnetic field**					
HMF_{center}	11.8 ± 1.3	50	/	/	/
GMF_{center}	11.3 ± 0.5	50	/	/	/
GMF	13.6 ± 0.6	50	/	/	/

Data are shown as mean ± SD from three measurements. IB_xI, component of |B| at the direction from South to North; IB_yI, from East to West; IB_zI, vertically downward. HMF_{center}, residue magnetic field at the center of the HCS under “zero” mode; $HMF_{avreage}$, average magnetic field of the central plane of the HCS at “zero” mode; $GMF_{center,}$ GMF at the center of the HCS when the power supplies were turned off; $GMF_{average}$/GMF, average GMF on the control table with HCS under “zero” mode.

Supplementary table S2. The primers used for qPCR assay.

Gene	primer	Sequence (5‘ to 3')	T_m (℃)	length (bp)
Tubulin 5α (Tubb5)	forward	TCACTGTGCCTGAACTTACC	58	318
	reverse	GGAACATAGCCGTAAACTGC		
Gapdh	forward	AGGTCGGTGTGAACGGATTTG	58	123
	reverse	TGTAGACCATGTAGTTGAGGTCA		
Nestin	forward	CCCACCTATGTCTGAGGCTC	58	166
	reverse	GGGCTAAGGAGGTTGGATCAT		
Sox2	forward	GCGGAGTGGAAACTTTTGTCC	62	157
	reverse	CGGGAAGCGTGTACTTATCCTT		
Gfap	forward	CCCTGGCTCGTGTGGATTT	60	238
	reverse	GACCGATACCACTCCTCTGTC		
ßIII-tubulin (Tubb3)	forward	TAGACCCCAGCGGCAACTAT	60	127
	reverse	GTTCCAGGTTCCAAGTCCACC		
Neurod1	forward	GCAGCTCTGGAGCCCTTCTT	58	190
	reverse	GCGGCACCGGAAGAGAAGAT		
Cry1	forward	CAGACTCTCGTCAGCAAGATG	62	204
	reverse	CAAACGTGTAAGTGCCTCAGT		
Cry2	forward	AGCACTTGGAACGGAAGG	62	140
	reverse	CAGGCGGTAGTAGAAGAGG		
